# Role of circulating tumour cells (CTCs) in recurrent/metastatic head and neck squamous cell carcinoma (HNSCC)

**DOI:** 10.3332/ecancer.2023.1578

**Published:** 2023-07-20

**Authors:** Annie Kanchan Baa, Atul Sharma, Suman Bhaskar, Ahitagni Biswas, Alok Thakar, Rajeev Kumar, Sreeja Jayant, Gourishankar Aland, Alain D’Souza, Vikas Jadhav, Atul Bharde, Jayant Khandare, Raja Pramanik

**Affiliations:** 1 Department of Medical Oncology, Dr B. R.A. Institute Rotary Cancer Hospital, All India Institute of Medical Sciences, New Delhi 110 029, India; 2 Department of Radiation Oncology, Dr B. R.A. Institute Rotary Cancer Hospital, All India Institute of Medical Sciences, New Delhi 110 029, India; 3 Department of Head and Neck Surgery, All India Institute of Medical Sciences, New Delhi 110 029, India; 4 Actorius Innovations and Research, Pune 411057, India, and Actorius Innovations and Research Co., Simi Valley, CA 93063, USA

**Keywords:** head and neck cancer, recurrent, biomarker, circulating tumour cells, response

## Abstract

**Background:**

Liquid biopsy is emerging as a non-invasive tool, providing a personalized snapshot of a primary and metastatic tumour. It aids in detecting early metastasis, recurrence or resistance to the disease. We aimed to assess the role of circulating tumour cells (CTCs) as a predictive biomarker in recurrent/metastatic head and neck cancer (head and neck squamous cell carcinoma (HNSCC)).

**Methodology:**

Thirty-five patients receiving palliative chemotherapy underwent blood sampling [2 mL in Ethylenediaminetetraacetic acid (EDTA) vial] at baseline and at 3 months intervals. The CTCs were isolated and evaluated using anti-epithelial cell adhesion molecule antibody-based enrichment using the OncoDiscover platform.

**Results:**

CTCs isolated from 80% of patients (*n* = 28) showed the sensitivity of cell detection at the baseline and 3 months intervals. The median CTC count was 1/1.5 mL of blood and the concordance with clinic-radiological outcomes was 51.4%. The median CTC count (1 (range:0–4) to 0 (range:0–1)) declined at 3 months in responders, while the non-responders had an increase in levels (0 (range :0–2) to 1 (range :0–3)). Although CTCs positively correlated with progression-free survival (PFS) and overall survival (OS), the association of CTCs did not show a significant difference with these parameters (PFS: 6 months versus 4 months; hazard ratio: 0.68; 95% confidence interval (CI): 0.29–1.58, *p* = 0.323; OS: 10 months versus 8 months; hazard ratio: 0.54; 95% (CI):0.18–1.57 *p* = 0.216) between CTC positive and CTC negative patients at 3 months.

**Conclusion:**

This study highlights the utility of CTC as a disease progression-monitoring tool in recurrent HNSCC patients. Our findings suggest the potential clinical utility of CTC and the need for exploration in upfront settings of the disease as well (NCT: CTRL/2020/02/023378).

## Introduction

Liquid biopsy has developed to be a quick, non-invasive tool capable of providing individualised information on primary and metastatic tumour [1]. Circulating tumour cells (CTCs), cell-free tumour DNA, proteins, metabolites, exosomes, mRNA and miRNAs are the frequently used analytes for liquid biopsy [2].

CTC was first described by Ashworth in 1869 as metastatic disease. CTCs are transient cancer cells that originate from a primary tumour or metastatic site with the capacity to enter the adjacent vasculature and cause dissemination to distant sites [3]. Also, they are extremely rare, with approximately 1–100 CTC/mL, making it challenging to detect and capture CTCs from whole blood samples [4]. However, its biophysical and biochemical properties have been extensively explored for selective isolation and characterisation. Size-based filtration and density gradient centrifugation are the methods that use the physical properties of CTCs for isolation, while biochemically, unique cancer-specific biomarkers are identified to ensure selective capture. Epithelial cell adhesion molecule (EpCAM), human epidermal growth factor receptor 2, folic acid receptor, and transferrin receptor are among the most extensively explored tumour-associated cell-surface antigens for immunomagnetic separation of CTCs.

Head and neck squamous cell carcinoma (HNSCC) is a heterogeneous group of cancers that are difficult to treat successfully. With dismal survival rates, they pose a substantial global health burden, despite advances in management [5, 6]. The substantial disease burden arises from Southeast Asian countries, with about one-third being contributed by India. Head and neck cancer assessment is principally done via clinical and radiological methods. Newer biomarkers aiding in the evaluation of HNSCC are an unmet need, as relapses are extremely common and often lead to aggressive disease. CTCs have emerged as a promising tool to diagnose, monitor and prognosticate a variety of epithelial cancer including HNSCC.

The ability of CTCs to steer chemotherapeutic decisions is being explored in phase III trials in solid malignancies (breast cancer: SWOG S0500, DETECT-III/IV; colon cancer VISNU-1) [7–9]. Initial efforts for liquid biopsies in head and neck cancer have shown encouraging results, particularly with nasopharyngeal carcinoma and human papilloma virus-positive HNSCCs [10, 11]. Studies have tried to establish CTCs as a positive biomarker in HNSCC [12]. Recently, Qayyumi *et al* [13] demonstrated the utility of preoperative CTCs to investigate aggressive clinicopathologic factors in the treatment-naïve oral squamous cell carcinoma patients. CTCs have also been analysed in HNSCC to assess the response to radiotherapy (RT) as well as chemotherapy aiding in prognostic stratification [14, 15].

In this study, we explored the clinical utility of CTCs to monitor treatment response in recurrent/metastatic HNSCC patients using the Drug Controller General of India (DCGI) approved OncoDiscover technology. CTCs were isolated using OncoDiscover technology that is based on EpCAM targeting immunomagnetic separation. CTCs-based liquid biopsy methods have enormous potential for improving early cancer detection, treatment modifications and surveillance for disease recurrence.

## Methods

### Study design and patient cohort

This was a single-centred, phase II interventional study conducted at All India Institute of Medical Sciences. Ethical approval from the ethical committee was granted via IECPG-755/30.01.2020 and the CTRI registration was taken (CTRI/2020/02/023378) before the commencement of the study. Patients of HNSCC (oral cavity, larynx, oropharynx and hypopharynx); having a recurrence of disease within 6 months of platinum therapy either in primary, recurrent or metastatic setting; having a radiologically measurable disease were considered eligible. Thirty-five patients were recruited at the Department of Medical Oncology, Head and Neck cancer clinic, AIIMS, New Delhi between January 2020 and December 2021, with a median follow-up of 8 months (95% CI:5–10.9 months). All the patients were administered chemotherapy as the intervention [Erlotinib/Methotrexate/5-Fluorouracil (EMF) regimen: erlotinib 150 mg once a day, methotrexate 40 mg/m^2^ (d1, d8) and 5-fluorouracil 500 mg/m^2^ (d1, d8)] every 28 days. Response assessment was done at 3 monthly intervals using contrast-enhanced computed tomography (CECT)-face/neck/chest and response was evaluated using RECIST 1.1. The primary objective was to assess the objective response rates of the regimen, which has been reported previously by our group [16]. Here we report the secondary objective of the study that was to assess the role of CTCs as a surveillance biomarker for disease recurrence and metastasis.

### Circulating tumour cells

For CTC isolation, 5 mL of venous blood sample [BD vacutainer-K2 Ethylenediaminetetraacetic acid (EDTA) 10.8 mg] was collected at baseline and post 3 months of therapy. The isolation and detection of CTCs were performed using OncoDiscover liquid biopsy technology, which has received regulatory approval from DCGI for CTC isolation technology. OncoDiscover technology works on the principle of EpCAM targeting immunomagnetic separation. It comprises a multi-component system consisting of glutathione-linked iron nanoparticles covalently conjugated to carbon allotropes like carbon nanotube (CNT) and graphene via the Poly amidoamine dendrimer as a linker. Finally, the iron NP–CNT substrate is crosslinked with an optimized amount of EpCAM antibodies via carbodiimide chemistry (WIPO patent no WO2016132265A2, 2016) [17, 18]. This technology has very high sensitivity, specificity and high negative predictive value [13]. CTCs were detected based on the presence of cytokeratin,18, a prominent epithelial marker, and the absence of CD45, a ubiquitous leukocyte marker expression. Cytokeratin 18 (CK18) and Cluster of differentiation 45 (CD45) expression was assessed using an immunofluorescence method with a combination of fluorescently labelled anti-cytokeratin and anti-CD45 antibodies for further characterization.

CTCs were defined as cells having CK18 expression, the presence of a prominent DAPI (4’,6-diaminido-2-phenylindol)-stained nucleus and the absence of CD45 expression, a prominent marker that defines leucocyte lineage. All detected CK18, DAPI positive and CD45 negative CTCs were reported into two categories: individual CTCs and CTC cluster (groups of two or more CTC seen together) per 1.5 mL of blood.

### Statistical analysis

Data analysis was done using IBM SPSS v.26 software package. Demographic and clinical characteristics were summarized (Table 1). Kaplan-Meier curves were constructed for the analysis of survival data, and the log-rank test was used for comparison. Univariate and multivariate survival analyses were performed using Cox proportional hazards model. *p* < 0.05 was considered statistically significant. A repeated measures test was used to check differences in responses with time and association with different variables.

## Results

### CTCs analysis

**Figure 1.**
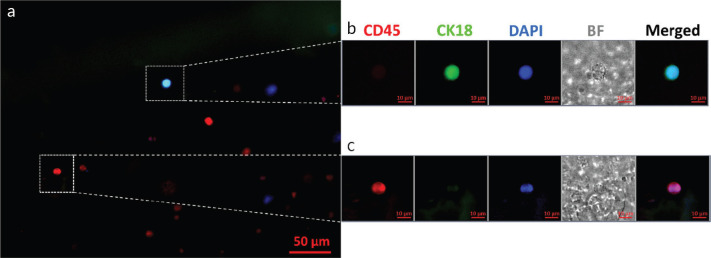
A representative fluorescence microscopy image of a CTC isolated from the peripheral blood of an oral cancer patient using OncoDiscover technology. (a): Merged image of CK18 expressing CTC (upper dotted white square) alongside few leukocytes expressing CD45 in the background. Scale bar is 50 μm. (b): Zoomed insight of CTC showing a cell with a prominent nucleus and strong expression of CK18. (c): Magnified region showing a leukocyte dimer with a strong CD45 expression. Scale bar is 10 μm.

CTCs from all positive samples showed well-defined cellular structures with CK18 expression, the absence of CD45 on the cell surface and a prominent nucleus. Figure 1 shows a representative fluorescence microscopy image of a CTC obtained from one of the patients at the baseline analysis using OncoDiscover technology. A low magnification image (zoomed out) showing a larger field of view (more than ~300 × 250 μm) has been elaborated. This image shows a CTC with few leukocytes in the background. Figure 1a shows CTC expressing CK18, upper dotted white square alongside few leukocytes expressing CD45 in the background (scale bar: 50 μm). Figure 1b highlights the zoomed image of CTC showing a cell with a prominent nucleus and strong expression of CK18. Figure 1c shows a magnified region showing a leukocyte dimer with a strong CD45 expression, but negative for CK18. (scale bar: 10 μm).

**Figure 2.**
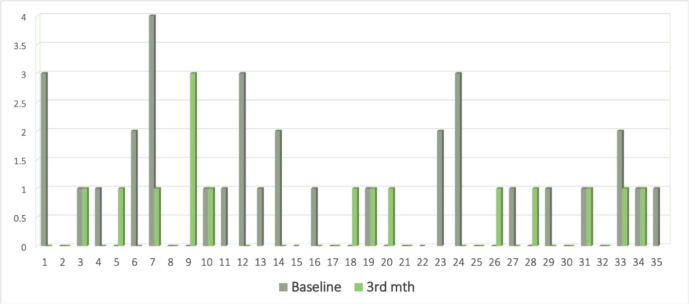
Bar graph showing CTC levels at 0 (grey) and 3 (green) months.

A baseline CTC analysis was performed for all 35 patients and detectable CTCs at baseline were observed in 20 (57.1%) patients while 15 (42.19%) cases did not show the presence of CTCs. The median CTC count among CTC-positive patients at the baseline was 1/1.5 mL of blood. Although we observed CTC clusters in our study population, they occurred at a moderate frequency (7 out of 35 patients, 20%) patients) and suggested the more aggravated state of the disease in the subpopulation from where they were detected. Comparable data at two time points (0 and 3 months) was available for 28 patients. The concordance and discordance rates with response were 51.4% (*n* = 18) and 11.4% (*n* = 4), respectively, while it was not evaluable for 37.1% (*n* = 13) of the patients (Figure 2). The median CTC count (1 (range:0–4) to 0 (range:0–1)) declined at 3 months in responders while the non-responders had an increase in levels (0 (range:0–2) to 1 (range:0–3)).

**Figure 3.**
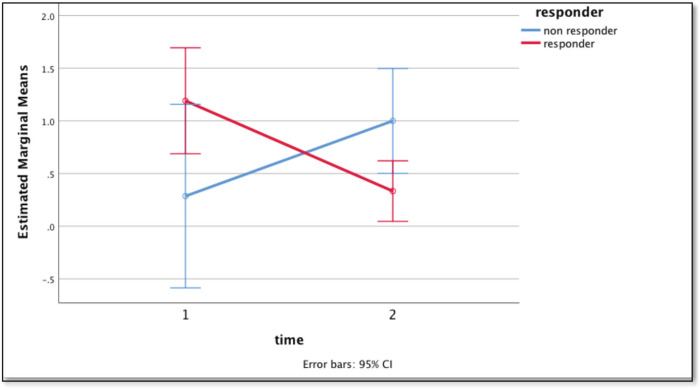
Graph showing the difference in mean CTC count with time: baseline (1) and at 3 months (2) using repeated measures test between the responders (red) and non-responders (blue).

Repeated time measures test showed a statistically significant difference (*p* = 0.010) in CTCs levels in responders and non-responders. The mean readings at the baseline for responders (*n* = 22) and non-responders (*n* = 6) were 1.19 ± 1.25 and 0.29 ± 0.488, respectively which reduced to 0.33 ± 0.48 and increased to 2 ± 0.01 in corresponding groups after the third month (Figure 3). Patients previously exposed to radiation therapy had a higher incidence of undetectable CTCs at baseline. However, this distinction was observational and did not correlate with statistical significance (S3, Table 2).

**Figure 4.**
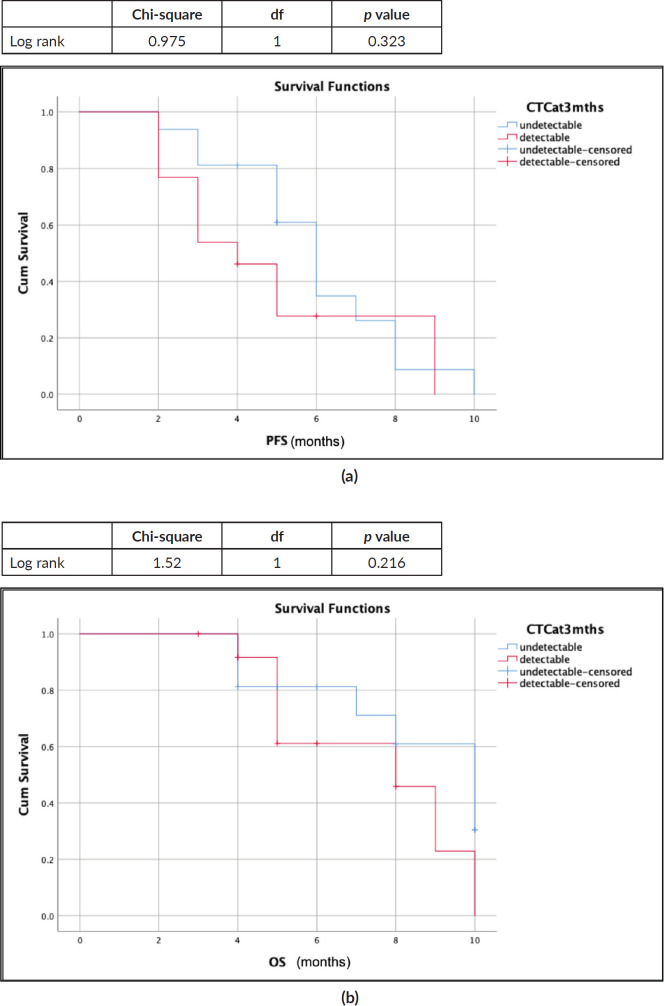
Survival outcomes (Kaplan-Meir curves) with CTC clearance at 3 months (blue: undetectable CTC; red: detectable CTC). (a): Median PFS: 6 months versus 4 months. (b): Median OS: 10 months versus 8 months.

Survival outcomes for patients with detectable CTCs at baseline and CTCs clearance at 3 months of EMF treatment were compared (S1, S2). The median progression-free survival (PFS) was 6 months (95% confidence interval (CI): 4.72–7.72) and 4 months (95% CI: 2.3–5.65); *p* = 0.323, while the median overall survival (OS) was 10 months (95% CI: 6.7–13.2) and 8 months (95% CI: 4.3–11.6); *p* = 0.216 for patients with undetectable and detectable CTCs at 3 months, respectively (Figure 4a and b). Although numerically better in those who cleared CTCs by the third month, there was no significant difference in survival (Table 2).

## Discussion

The exciting investigational avenue of CTCs has attracted researchers to explore the potential use of CTCs as a predictive and prognostic marker. To this end, a variety of clinical studies have investigated the translational benefits of CTCs in HNSCC. Jatana *et al* [19] prospectively followed 48 HNSCC patients and measured CTC numbers by the negative depletion method to assess the CTC dynamics. An improved disease-free survival (DFS) was seen with a reduction of CTC number and a worse clinical outcome with >25 CTCs/mL. However, in a large cohort study of 144 patients with locally advanced HNSCC and administered with adjuvant chemotherapy postoperatively, Tinhofer *et al* [20] investigated the utility of CTC as a monitoring tool. This study suggested that the presence of CTCs was not predictive of DFS and OS in the cohort. Both studies had taken samples only at a one-time point and assessed the survival outcome. Following up on CTCs at baseline and post-intervention would be valuable for better prognostication.

Our study reports CTC as a biomarker in recurrent/metastatic HNSCC, which is a difficult scenario but an unmet need. The detailed characteristics of the patients recruited have been elaborated in our previous study [16]. The CTCs clearance at 3 months of palliative chemotherapy (EMF: erlotinib + methotrexate + 5-Flourouracil) has been associated with a better PFS (6 months versus 4 months) and OS (10 months versus 8 months) numerically. The decline in CTC count synchronizes with the response assessed radiologically (Figure 2) which was statistically significant (*p* = 0.010).

Inhestern *et al* [21] evaluated CTCs in locally advanced oral and oropharyngeal squamous cell cancer while Wang *et al* [22] assessed the same in metastatic breast cancer. Both studies inferred that changes in CTC levels were highly correlative with tumour response to treatment. Our observations on CTC levels in HNSCC corroborate well with these studies. The persistently high CTC levels were associated with worse prognosis and treatment response and decreasing CTC levels correlated with improved response and outcomes [21, 22]. Kulasinghe *et al* [11] and Kong *et al* [2] demonstrated higher CTC count and the presence of CTC clusters was significantly associated with distant metastatic disease and higher nodal burden in head and neck cancer.

The heterogeneity of the disease is manifested by variations in the detection rates. Qayyumi *et al* [13] assessed 152 treatment naïve oral squamous cell carcinoma patients along with 40 controls yielding a sensitivity of 94.3%, specificity of 98% and accuracy of 95.17%. The cut-off for true positives was taken as 3.5 CTC/1.5 mL; CTCs >20, and >13 suggested nodal and advanced disease respectively [13]. This study further demonstrated CTC as an independent factor to predict disease progression and OS. Additionally, the findings open the possibility of investigating CTCs as a diagnostic tool, as CTCs were also detected in early-stage patients at the time of disease presentation. Zhou *et al* [22] demonstrated a CTC positivity rate of 81% with a median CTC of 7.5/7.5 mL (range:0–13) in 95 upfront HNSCC patients. Liu *et al* [15] reported a mean CTC count of 2/3.2 mL blood on analyzing 178 patients.

The detection rates of CTC and CTC clusters were quite low in our study; despite the heavy burden of the disease. The median baseline CTC was 1/1.5 mL with a 60% positivity rate.

This may be attributed to the various factors of having a small sample size (*n* = 35), a single-arm study without any controls. The setting was different – we had a recurrent/metastatic cohort in contrast to treatment-naïve patients in the aforementioned studies. The comparison was difficult with baseline negative results of CTC. The patients with previous exposure to RT had higher rates of undetectable CTCs at baseline, however, it was not statistically significant. Our analysis was limited due to the smaller cohort of patients and low detection rates, despite the advanced stage of the disease.

Non-invasive markers are an unmet need in HNSCC owing to their aggressive nature and increased recurrence rates. Our study reports a biomarker in relapsed/metastatic HNSCC, which has been a difficult disease to tackle in low-income populations. In such situations, CTC can be an excellent disease monitoring and surveillance tool.

## Conclusion

Our study highlights the role of CTCs as a biomarker in recurrent/metastatic head and neck squamous cell carcinoma which is a global challenge. This will help to strengthen the armamentarium for better management of the aggressive pathology claiming lives.

## Conflicts of interest

The authors have no conflict of interest.

## Financial disclosure

OncoDiscover sponsored the analysis of CTCs.

## Consent to participate

Informed consent was obtained from all patients.

## Ethical approval

The study protocol was approved by the Institute Ethics Committee vide letter number: IECPG-755/30.01.2020.

## Availability of data and material

Data regarding this study will be available from the corresponding author (RP) on reasonable request.

## Author contributions

Study concept/study design/data acquisition: RP/AS/AKB

Quality control of data and algorithm: AKB/RP

Data analysis and interpretation: RP/AKB

Statistical Analysis: AKB/RP

Manuscript preparation: AKB/RP

Manuscript editing/review: RP/AS/AKB/AB/SB/AT/RK/JK/AD/GS/ABh/SJ/VK

The study abstract was accepted in ASCO 2022 for online publication.

## Figures and Tables

**Table 1.** Baseline characteristics.

Variables	*N* (%)
Age, median and range (years)	45 (26–65 years)
Sex - Male - Female	28 (80%) 7 (20%)
Smoking/tobacco - Yes - No	33 (94.3%) 2 (5.7%)
Primary site - Buccal mucosa - Lateral border of the tongue - Base of tongue - Others	22 (62.9%) 3 (8.6%) 5 (14.3%) 5 (14.3%)
Extent - Locoregional - Local and distant metastasis (lungs)	30 (85.7%) 5 (14.2%)
Lines of previous therapy - 1 - >1	34 (97.1%) 1 (0.02%)
Previous radiation - Yes - No	21 (60%) 14 (40%)

**Table 2.** PFS and OS correlation with CTC clearance at 3 months.

CTCs at 3 months	Median PFS months (95% CI)		Median OS months (95% CI)	
Undetectable	6 (4.99–7.01)	*p* = 0.323	10 (7.8–12.1)	*p* = 0.216
Detectable	4 (2.3–5.65)		8 (4.3–11.6)	

## References

[1] Singhal A, Hussain A, Agarwal A (2019). Current status of cell-free DNA in head and neck cancer management. Ann Indian Acad Otorhinolaryngol Head Neck Surg.

[2] Kong L, Birkeland AC (2021). Liquid biopsies in head and neck cancer: current state and future challenges. Cancers (Basel).

[3] Pantel K, Brakenhoff RH, Brandt B (2008). Detection, clinical relevance and specific biological properties of disseminating tumour cells. Nat Rev Cancer.

[4] Ming Y, Li Y, Xing H (2017). Circulating tumor cells: from theory to nanotechnology-based detection. Front Pharmacol.

[5] Noronha V, Joshi A, Marfatia S (2016). Health-related quality of life in patients with metastatic, relapsed, or inoperable squamous cell carcinoma of the head and neck in India. Support Care Cancer.

[6] Sung H, Ferlay J, Siegel RL (2021). Global cancer statistics 2020: GLOBOCAN estimates of incidence and mortality worldwide for 36 cancers in 185 countries. CA Cancer J Clin.

[7] Paoletti C, Miao J, Dolce EM (2019). Circulating tumor cell clusters in patients with metastatic breast cancer: a SWOG S0500 translational medicine study. Clin Cancer Res.

[8] Krause S, Friedl T, Romashova T (2019). Abstract OT1–10-01: DETECT III/IV study trial – the multicenter study program in patients with HER2-negative metastatic breast cancer and circulating tumor cells. Cancer Res.

[9] Aranda E, Viéitez JM, Gómez-España A (2020). FOLFOXIRI plus bevacizumab versus FOLFOX plus bevacizumab for patients with metastatic colorectal cancer and ≥3 circulating tumour cells: the randomised phase III VISNÚ-1 trial. ESMO Open.

[10] McMullen KP, Chalmers JJ, Lang JC (2016). Circulating tumor cells in head and neck cancer: a review. World J Otorhinolaryngol Neck Surg.

[11] Kulasinghe A, Tran THP, Blick T (2017). Enrichment of circulating head and neck tumour cells using spiral microfluidic technology. Sci Rep.

[12] D’Souza A, Hossain MM, Jayant S (2021). Circulating tumor cells demonstrate a positive biomarker in head and neck squamous cell carcinoma (HNSCC) in tobacco consuming population of Bangladesh.

[13] Qayyumi B, Bharde A, Aland G (2022). Circulating tumor cells as a predictor for poor prognostic factors and overall survival in treatment naïve oral squamous cell carcinoma patients. Oral Surg Oral Med Oral Pathol Oral Radiol.

[14] Perumal V, Corica T, Dharmarajan AM (2019). Circulating tumour cells (CTC), head and neck cancer and radiotherapy; future perspectives. Cancers (Basel).

[15] Liu K, Chen N, Wei J (2020). Clinical significance of circulating tumor cells in patients with locally advanced head and neck squamous cell carcinoma. Oncol Rep.

[16] Baa AK, Sharma A, Bhaskar S (2022). A single-arm feasibility phase II study of EMF (erlotinib + methotrexate + 5-fluorouracil) regimen in platinum-refractory recurrent/metastatic head and neck squamous cell carcinoma (R/M HNSCC). Ecancermedicalscience.

[17] Khandare JJ, Banerjee S, Padigaru M (2016). Multifunctional magneto-polymeric nanosystems for rapid targeting, isolation, detection and simultaneous imaging of circulating tumor cells.

[18] Singh B, Arora S, D’Souza A (2021). Chemo-specific designs for the enumeration of circulating tumor cells: advances in liquid biopsy. J Mater Chem B.

[19] Jatana KR, Balasubramanian P, Lang JC (2010). Significance of circulating tumor cells in patients with squamous cell carcinoma of the head and neck: initial result. Arch Otolaryngol Neck Surg.

[20] Tinhofer I, Konschak R, Stromberger C (2014). Detection of circulating tumor cells for prediction of recurrence after adjuvant chemoradiation in locally advanced squamous cell carcinoma of the head and neck. Ann Oncol.

[21] Inhestern J, Oertel K, Stemmann V (2015). Prognostic role of circulating tumor cells during induction chemotherapy followed by curative surgery combined with postoperative radiotherapy in patients with locally advanced oral and oropharyngeal squamous cell cancer. PLoS One.

[22] Wang C, Mu Z, Chervoneva I (2017). Longitudinally collected CTCs and CTC-clusters and clinical outcomes of metastatic breast cancer. Breast Cancer Res Treat.

[23] Zhou S, Wang L, Zhang W (2021). Circulating tumor cells correlate with prognosis in head and neck squamous cell carcinoma. Technol Cancer Res Treat.

